# Critically ill patients with cancer and sepsis: clinical course and prognostic factors

**DOI:** 10.1186/cc10158

**Published:** 2011-06-22

**Authors:** LSCF Rabello, M Rosalem, T Lisboa, P Caruso, R Costa, J Leal, J Salluh, M Soares

**Affiliations:** 1 Instituto Nacional de Câncer, Rio de Janeiro - RJ, Brazil; 2 Hospital de Clínicas, Universidade Federal do Rio Grande do Sul, Porto Alegre - RS, Brazil; 3 Hospital A. C. Camargo, São Paulo - SP, Brazil; 4 D'Or Institute for Research and Education, Rio de Janeiro - RJ, Brazil

## Introduction

Sepsis is a frequent complication in patients with cancer associated with adverse outcomes. The aim of this study was to evaluate the clinical course and to identify independent predictors of mortality in these patients.

## Methods

We performed a secondary analysis of a prospective cohort study conducted at an oncological medical-surgical ICU. Logistic regression was used to identify predictors of hospital mortality.

## Results

A total of 563 patients (77% solid tumor; 23% hematological malignancies) were included over a 55-month period. The most frequent sites of infection were the lung, abdomen and urinary tract; 91% patients had severe sepsis/septic shock. Gram-negative bacteria were responsible for more than half of the episodes of infection; 207 (38%) patients had polymicrobial (>1 infectious agent) infections. ICU, hospital and 6-month mortality rates were 51%, 65% and 72%. In multivariate analyses, sepsis in the context of medical complications, active disease, compromised performance status, presence of three or four SIRS criteria, and the presence of respiratory, renal and cardiovascular failures were associated with increased mortality. Adjusting for other covariates, patients with urinary tract infection had better outcomes. Patients could be stratified into categories of risk for death according to the number of clinical predictors.

## Conclusion

Our results can be of help to assist intensivists in clinical decisions and counseling of patients and families, and to contribute with future research to improve characterization and risk-stratification in these patients.

